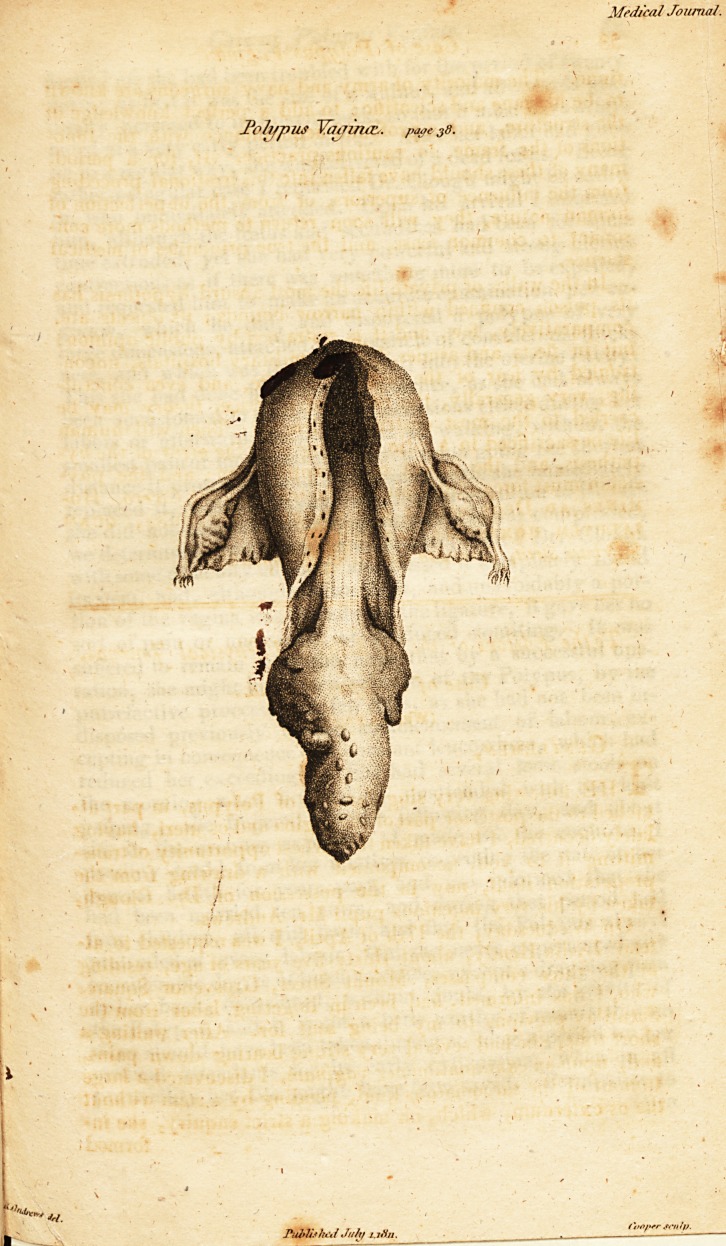# Case of Polypus Vaginæ

**Published:** 1811-07

**Authors:** E. P. Fordham

**Affiliations:** South Audley Street


*To the Editors of the Medical and Physical Journal.*

### *Case of Polypus Vaginæ.*

(With an Engraving.)

GENTLEMEN,

THE following very singular case of Polypus, in part attached to the posterior part of the vagina and os uteri, having lately occurred, I have taken the earliest opportunity of transmitting it to you, accompanied with a drawing from the preparation itself, now in the possession of Dr. Clough, taken by his very ingenious pupil Mr. Andrews.

On Wednesday, the 17th of April, I was requested to attend Lydia Hendy, about thirty-five years of age, residing at the three compasses, Mount Street, Grosvenor Square, who, I was informed, had been in lingering labor from the Saturday previous to my being sent for. After waiting a short time, she had several very strong bearing down pains, and, upon an examination *per vaginam*, I discovered a large tumour of the sarcomatous kind, pending by a stem without the os externum, which, on making a strict enquiry, she informed

*Polypus Vaginæ.* page 38.

[illegible] Andrews del.

Published July 1, 1811.

Cooper sculp.

formed me she had been troubled with for the period of twenty years; at first it was the size of a walnut, and afterwards gradually increased to its present bulk, so that it must have originated at a very early period of life. After her labor had gone on in a regular way, she was delivered of a dead fœtus. Being rather a singular case I requested Dr. Clough might be called in, who immediately attended. Upon his arrival I informed him, although the fœtus and secundines had been for some time extruded, yet she had very powerful and strong uterine contractions as if there was something more to be expelled, and requested him to make an accurate examination *per vaginam*, which he did, and discovered a polypus of very large dimensions, attached by a pedicle of considerable thickness, and which was then pushed up into the uterine cavity. This she had done herself in my absence, as she had always been accustomed to do on former occasions either during her labors or afterward, or in the act of walking, without the smallest pain or inconvenience: even upon going the shortest distance it protruded, and by stepping aside she immediately replaced it, without paying any further attention to it. As she did not complain of any pain when it was compressed, we determined upon passing a ligature, which Dr. Clough with some difficulty effected, and passed it completely round its stem, and, although drawn tight, and unavoidably a portion of the vagina was included in the ligature, it gave her no sort of pain or uneasiness, or produced vomiting. It was suffered to remain with the hope that by a successful operation, she might have been released of the Polypus, by the putrefactive process, in a few days, as she had not been indisposed previously to the commencement of labour, excepting in consequence of a constant leucorrhœa, which had reduced her exceedingly. She had several loose stools on the morning when first taken, unattended with the least griping; she lingered till the following day, and about six in the evening expired. I made all the enquiries I possibly could from her relatives respecting the state of her general health for years past, and was informed that she had been married ten years, and during that period had four children, all still born, and that the Polypus always protruded at every labour; that she never experienced any considerable degree of inconvenience, except a dragging pain in her back, occasioned, without doubt, by the gravity of the Polypus, and it is not a little worthy of remark that no steps should have been taken during so long a period by her medical attendants for its early extirpation, when in all probability her life might have been saved. Permission to open open the body being obtained, it was performed by Mr. Chevalier in the presence of Dr. Clough, Mr. Kitching, and myself. The polypus was found, as before described, in the posterior part of the vagina; no particular disease existing in the uterus or its appendages: there was a slight degree of inflammation in the colon and larger intestines. The uterus and appendages with the polypus were dissected out, and an incision made into the latter, in order to discover its true texture. In addition to the above particular, I beg leave to state, I feel much indebted to my friend, Dr. Clough, for his promptitude on this occasion, and much pleasure in having an opportunity of putting him in possession of so valuable a morbid preparation, in addition to his extensive collection. Dr. Clough considered the Polypus to be the largest he ever saw, excepting two, which were in the late Dr. W. Hunter's Museum; observing that its appearance was very similar to that of the excrescence from the fundus uteri, with an inversion, of which Dr. Denman has given a delineation in his series of engravings on this subject. It measured from the beginning of the stem to the apex eight inches, seven inches and a half in circumference, three inches in the thickest part, and in weight was one pound four ounces.

I remain, Gentlemen,

Your most obedient Servant,

E. P. FORDHAM.

*South Audley Street, May* 7, 1811.

## Figures and Tables

**Figure f1:**